# Clinical Outcome and Management for Geriatric Traumatic Injury: Analysis of 2688 Cases in the Emergency Department of a Teaching Hospital in Taiwan

**DOI:** 10.3390/jcm7090255

**Published:** 2018-09-04

**Authors:** Meng-Yu Wu, Yu-Long Chen, Giou-Teng Yiang, Chia-Jung Li, Amy Shu-Chuan Lin

**Affiliations:** 1 Department of Emergency Medicine, Taipei Tzu Chi Hospital, Buddhist Tzu Chi Medical Foundation, New Taipei 231, Taiwan; skyshangrila@gmail.com (M.-Y.W.); yulong0129@gmail.com (Y.-L.C.); gtyiang@gmail.com (G.-T.Y.); 2 Department of Emergency Medicine, School of Medicine, Tzu Chi University, Hualien 970, Taiwan; 3 Research Assistant Center, Show Chwan Memorial Hospital, Changhua 500, Taiwan; 4 Department of Medical Research, Chang Bing Show Chwan Memorial Hospital, Changhua 505, Taiwan; 5 Superintendent Office, Nursing Department, Show Chwan Memorial Hospital, Changhua 505, Taiwan; 6 College of Nursing and Health Sciences, Da-Yeh University, Changhua 515, Taiwan

**Keywords:** geriatric population, traumatic injury, shock index, mortality

## Abstract

Geriatric traumatic injuries in emergency departments are frequent and associated with higher mortality rates and catastrophic functional outcomes. Several prediction scores have been established to manage traumatic patients, including the shock index (SI), revised trauma score (RTS), injury severity score (ISS), trauma injury severity score (TRISS), and new injury severity score (NISS). However, it was necessary to investigate the effectiveness and efficiency of care for the geriatric traumatic population. In addition, image studies such as computed tomography and magnetic resonance imaging play an important role in early diagnosis and timely intervention. However, few studies focus on this aspect. The association between the benefit of carrying out more image studies and clinical outcomes remains unclear. In this study, we included a total of 2688 traumatic patients and analyzed the clinical outcomes and predicting factors in terms of geriatric trauma via pre-hospital and in-hospital analysis. Our evaluation revealed that a shock index ≥1 may be not a strong predictor of geriatric trauma due to the poor physical response in the aging population. This should be modified in geriatric patients. Other systems, like RTS, ISS, TRISS, and NISS, were significant in terms of predicting the clinical outcome.

## 1. Introduction

Geriatric traumatic injuries frequently present in emergency departments and are always associated with catastrophic functional outcomes [1,2]. The higher mortality rate was reported in a previous study to be due to age-related factors, including comorbidity, poor physical reserves and the function of systemic compensation [3]. Age-related comorbidities may impair the insufficient physical reserves of cardiac output, vascular tone regulation, and sympathetic system balance [4,5]. The symptoms and signs of hemodynamic change are not easily detected due to the poor response in terms of peripheral vascular resistance and a lower maximum heart rate. Atherosclerosis in the aging population is another reason for the poor vascular response and atypical presentation [6]. A study including more than 6000 accident cases revealed that the mortality rate compared to the younger group was about 10% higher in the 70 years and older age group [7]. Similar results were also found in 111 United States and Canadian trauma centers [8]. The atypical presentation in geriatric traumatic patients is a challenge for physicians to diagnose shock. For early diagnosis and timely intervention for shock, more imaging studies would need to be arranged. However, the benefit of more imaging studies for the geriatric traumatic population remains unclear. In addition, several prediction score have also been promoted to manage traumatic patients, including the shock index (SI), revised trauma score (RTS), injury severity score (ISS), trauma injury severity score (TRISS), and new injury severity score (NISS). These scores were commonly used to survey traumatic patients and provided an early warning sign for physicians [9,10,11,12]. The shock index, defined as the ratio of the heart rate and systolic blood pressure, was promoted as a simple clinical tool that allows for rapid risk stratification without sophisticated calculations. Several studies have demonstrated the shock index as a useful predictor tool for hospital mortality in adult trauma patients [13,14,15]. However, the effectiveness, efficiency and suitability of these prediction scores in the geriatric population have been little analyzed. In this study, we included 2688 Taiwanese traumatic patients and investigated the pre-hospital and in-hospital data to analyze clinical outcomes and the prediction scores for the elderly and younger patient groups. 

## 2. Patients and Methods

### 2.1. Patients

**Figure 1:**
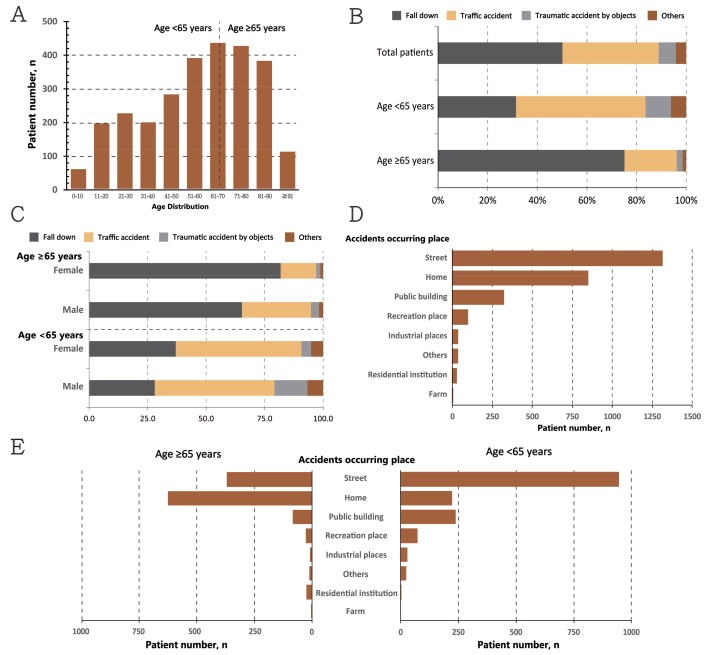
Clinical characteristics of the 2688 traumatic patients: (**A**) Age distribution in total patients; (**B**) The reasons for emergency admission in total patients and both groups; (**C**) The reasons for emergency admission with gender distribution; (**D**) The accidents occurring place in total patients; (**E**) The place of accidents in both groups.

This retrospective descriptive study was performed in Taipei Tzu Chi Hospital from January 2016 to March 2018 by Taipei Tzu Chi Hospital, Buddhist Tzu Chi Medical Foundation, New Taipei and approved by the Institutional Review Board of Taipei Tzu Chi Hospital (IRB number: 07-X-078). We included all traumatic patients from the trauma database. The inclusion criteria included traumatic patients from January 2016 to March 2018 who visited Taipei Tzu Chi Hospital and had a hospitalization history. Some patients received outpatient department follow-up; the exclusion criteria were the patients without hospitalization. In total, 2688 patients met the criteria and were included in the database. In our study, the age distribution of the included patients is shown in Figure 1A. The age range was from 0 to 101 years. To decrease the selective bias, we did not exclude patients of an age <18. The detailed demographics, overall survival and clinical outcome data were collected from the trauma database, computerized records, and charts. The pre-hospital collected data included age and sex, comorbid conditions, injury location, type of injuries, pre-hospital vital signs, and EMT treatment. The in-hospital parameters included triage, trauma team activation, in-hospital vital signs, and emergent treatment. The New Trauma and Injury Severity Score (TRISS), Injury Severity Score (ISS), New Injury Severity Score (NISS) and Revised Trauma Score (RTS) were also collected. The clinical outcome was analyzed via hospitalization time, intensive care unit (ICU) admission, ICU re-admission, ICU admission time, operation, re-operation and mortality.

### 2.2. Statistical Analysis

The detail demographic, overall survival and clinical outcome data related to elderly (age ≥65 years) and younger (age < 65 years) groups were analyzed using Chi-square analysis and independent sample *t*-test in the SPSS software (Version 13.0 SPSS Inc, Chicago, IL, USA) for statistical analysis. The association between the clinical parameters and clinical outcomes in the younger and elder groups was assessed by logistic regression. Statistical significance was defined as a *p*-value < 0.05.

## 3. Results

### 3.1. Patient Characteristics and Pre-Hospital Analysis

The characteristics of the total traumatic patients were shown in Figure 1 and analyzed in Table 1. There were 2688 patients included with a mean age of 57.1 ± 23.4; 1420 (52.8%) patients were male. In total, 1150 (42.8%) patients were aged ≥65 and 1538 (57.2%) patients were aged <65. In those aged <65 years, most patients were male (953 patients, 62%, *p* < 0.001). The age distribution was “M-shaped” for the total patients with two peaks at 61–70 and 21–30 age (Figure 1A). The reasons for emergency admission in the total patients were analyzed and revealed that falling accidents accounted for about 50.1%, followed by traffic accidents (38.8%), objects accidents (6.9%), including contusion and cutting, and others (4.1%), such as explosion injuries and gun shots. In the younger group, traffic accidents was major reason for emergency admission, with up to 52.1%, followed by falling (31.4%), contusion accidents with objects (10.3%), and others (6.1%). In the age group ≥65 years, falling accidents was a major reason for hospital admission, accounting for up to 75.1%, followed by traffic accidents (21.0%), contusion accidents with objects (2.4%), and others (1.4%) (Figure 1B). The gender distribution in both groups was analyzed and revealed that elder females had a greater risk of falling down and the younger male group had a greater risk of admission for traumatic accidents with objects (Figure 1C). In terms of the place accidents occurred, the street was the major place where accidents occurred for the total patients, especially for the younger group compared to the elderly group (Figure 1D,E). The arrival time of an emergency medical technician (EMT) was 11.2 ± 12.8 and the prehospital cardiac arrest rate was 2.1%. The pre-EMT time was not significantly different between the two groups. For prehospital cardiac arrest, the age group <65 years had a higher incidence rate (49 patients, 3.2% vs. 8 patients, 0.7%). In terms of pre-hospital vital signs, higher systolic blood pressure (SBP) and diastolic blood pressure (DBP) was noted in the elderly group with a lower heart rate (HR) and respiratory rate (RR). The consciousness status was alert for 2427 (90.3%) patients, voice and pain for 133 (5.0%) and unresponsive for 127 (4.7%) patients. In the younger group, the rate of unresponsive conscious status was higher (*p* < 0.001). The shock index was 0.65 ± 0.28 for the total patients and the younger group had a higher shock index compared to the elderly group. With regards to EMT treatment, the proportion of oxygen and laryngeal mask airway (LMA) used was higher for the age group <65 years. The same results were noted for cardiopulmonary resuscitation (CPR).

### 3.2. In-Hospital Analysis

The triage analysis found that the younger group had significantly more stage I triage compared to the elderly group. A similar result was noted for trauma team activated. With regard to in-hospital vital signs, higher SBP, DBP and RR was found for the elderly group. The shock index was higher in <65 years group, but the elderly group had more patients with a shock index >1. The comorbidity analysis found that the elderly patients had more cerebrovascular disease and coronary artery disease, but younger group had more metabolic disease. In terms of emergency treatment, the proportion of endotracheal tubes used was higher in the group aged <65 years. The results of the in-hospital analysis for the total traumatic patients are shown in Table 2.

### 3.3. Clinical Image Study Analysis and Outcome Analysis

In terms of image studies, head and neck computed tomography (CT) was most commonly used to rule out intracranial lesions and whole body CT was more frequently used in the <65 years group. Magnetic resonance imaging and angio-intervention was less commonly used. The results of the clinical image study analysis for the total traumatic patients are shown in Table 3. The New Trauma and Injury Severity Score (TRISS), Injury Severity Score (ISS), New Injury Severity Score (NISS) and Revised Trauma Score (RTS) were not significant. In the clinical outcome analysis, the ICU admission and ICU re-admission rate, ICU admission time, operation rate, re-operation rate, and mortality rate were not significant, as seen in Table 4.

### 3.4. Clinical Prediction Score Analysis

The association between the clinical parameters and clinical outcomes in the younger and elderly groups was assessed using logistic regression. The gender distribution significantly impaired the clinical outcome only in <65 years group. In the pre-hospital analysis, the diastolic blood pressure was found to be significant, except for the elderly group. The shock index and shock index >1 were not significant for the total patients and the subgroups. The current prediction systems, including the New Trauma and Injury Severity Score (TRISS), Injury Severity Score (ISS), New Injury Severity Score (NISS) and Revised Trauma Score (RTS) were significant (Table 5). 

## 4. Discussion

With an increasingly aging population, geriatric trauma will continue to increase. The delayed physical response to injury in the aging population makes it difficult to distinguish the best triage and management options. In previous studies, advanced age is a reported risk factor predicting poor clinical outcomes in traumatic injuries [16]. Aggressive management and intensive monitoring are necessary in the geriatric traumatic population, including aggressive resuscitation and more image studies. The image study analysis found that the CT images were more significantly used for the younger group than for the elderly group, especially head and neck CTs and whole body CT scans. This is compatible with the injury type in the younger group, for which traffic accidents was a major cause of injury. The univariate analysis showed the association with mortality was not significant in both groups. There are several reasons that could explained this result. First, an early CT scan may not detect early internal bleeding and intracranial hemorrhage. Second, keeping the stable hemodynamic condition required for a CT scan in critical patients is difficult. This may impair the results. In our experience, a CT scan is necessary for critical traumatic patients, but the progression in the hemodynamic condition limits the CT scan.

Few studies have analyzed the pre-hospital data of the geriatric traumatic population to predict clinical outcomes. In our data, the pre-hospital cardiac arrest rate was higher in the younger group, with lower SBP and higher HR. The pre-hospital vital signs were significantly different; in the elderly group, a lower SBP/DBP and higher RR/HR was noted. In the logistic regression analysis, the DBP was significant for the total patients (odds ratio: 0.981, CI: 0.996–0.995, *p*: 0.018) but not significant in the subgroups. Compared to the geriatric population, the shock index was higher in the elderly group. In the study by Viraj Pandit et al. [17], the mean shock index was 0.58 and a shock index ≥1 was noted in 3% of patients. They revealed that a shock index ≥1 was the strongest predictor of mortality with an odds ratio of 3.1. Our study showed that the shock index in both groups was significant, not only in the pre-hospital analysis but also in the hospital data. The aging population had a lower shock index but more patients with a shock index ≥1. The logistic regression analysis found that the shock index and a shock index >1 were not significant in terms of predicting mortality. The shock index might be impaired by poor physical reserves, systemic inflammation, and vascular tone regulation [18]. The comorbidity analysis found that coronary artery disease was a major diseases in the aging population, followed by metabolic disease and cerebrovascular disease. In the younger group, a much greater injury severity was noted and received more pre-hospital management. This result was supported by triage (*p* < 0.001) and trauma team activation (*p* < 0.001). In a previous study it was found that older adults, even with a lower injury severity, are hospitalized for injury more often than younger adults [19]. We investigated several clinical outcomes and revealed no significant difference in both groups. In our analysis, older adults with a lower injury severity may present the same clinical outcome as the younger group.

Analysis of the aggressive management revealed that computed tomography (CT) was mostly used for traumatic injuries to manage the injury site and internal bleeding [20,21]. Magnetic resonance imaging is another tool to survey spinal cord injuries and brain trauma [22,23]. The incidence of traumatic injuries in patients over 65 years of age is greater than in younger populations, even from low-energy injuries. In a FINE study [24], the NEXUS algorithms and decision rules were not found to be a valid tool to rule out injury. In geriatric trauma, computed tomography was usually used to rule out intracranial hemorrhage, even for low-energy trauma. In our analysis, head and neck CT was the most commonly used in both groups. The younger population received more CT scans compared to the geriatric traumatic population. In RTS, ISS, TRISS, and NISS, the CT scan was not significant in the total patients and subgroup analysis. Four common traumatic scores predicting the mortality rate were analyzed, including RTS, ISS, TRISS, and NISS. The scores were not significantly different for both groups. In RTS, ISS, TRISS, and NISS, these scores were significant for geriatric traumatic injuries. The clinical outcomes, including hospital time, intensive care unit (ICU) admission rate, ICU re-admission rate, ICU admission time, operation rate, re-operation rate, and mortality rate, were similar in both groups. Compared to previous studies, the results revealed that advanced age is not a significant predicting factor of the mortality rate [25,26]. 

Our study had some limitations. The retrospective study design makes it difficult to establish causality and the small sample size was another limitation. However, it provides a clear snapshot of clinical progression in the geriatric population. Future studies should explore lobotomy data and other factors contributing to the clinical outcomes to provide a strong foundation for developing a prediction score in the geriatric traumatic population.

## 5. Conclusions

In conclusion, the findings of this study significantly increase the understanding of the clinical outcomes and predicting factors in geriatric trauma via pre-hospital and in-hospital analyses. Our evaluation revealed that a shock index ≥1 may be not be a strong predictor in geriatric trauma due to the poor physical response of the aging population. This should be modified in geriatric patients. Other systems, like RTS, ISS, TRISS, and NISS, were significant in terms of predicting the clinical outcome.

## Figures and Tables

**Table 1:** Clinical characteristics of the total patients.

Characteristics	Total Patient	Age ≥ 65 Years	Age < 65 Years	*p*-Value
Patient number, *n* (%)	2688	1150 (42.8)	1538 (57.2)	
Age (years), mean ± SD	57.1 ± 23.4	78.7 ± 8.6	41.0 ± 17.0	*p* < 0.001
Gender, *n* (%)				*p* < 0.001
Female	1268 (47.2)	683 (59.4)	585 (38.0)	
Male	1420 (52.8)	467 (40.6)	953 (62.0)	
Pre-EMT time, mean ± SD	11.2 ± 12.8	11.8 ± 11.8	10.8 ± 14.0	0.178
Pre-hospital cardiac arrest, *n* (%)	57 (2.1)	8 (0.7)	49 (3.2)	*p* < 0.001
Pre-hospital vital sign, mean ± SD				
SBP	142.0 ± 29.1	154.9 ± 28.6	132.5 ± 25.6	*p* < 0.001
DBP	82.5 ± 17.9	84.0 ± 17.5	81.5 ± 18.0	0.014
RR	18.1 ± 2.4	17.9 ± 2.1	18.2 ± 2.6	0.007
HR	87.2 ± 17.5	84.5 ± 16.9	89.1 ± 17.7	*p* < 0.001
Conscious status, *n* (%)				
Alert	2427 (90.3)	1048 (91.1)	1379 (89.7)	0.211
Voice, pain	133 (5.0)	40 (3.4)	93 (6.0)	0.233
Unresponsive	127 (4.7)	61 (5.5)	66 (4.3)	*p* < 0.001
Shock index, mean ± SD	0.65 ± 0.28	0.57 ± 0.16	0.70 ± 0.33	*p* < 0.001
EMT treatment, *n* (%)				
O2 use	155 (5.8)	42 (3.7)	113 (7.3)	0.002
LMA use	36 (1.3)	5 (0.4)	31 (2.0)	*p* < 0.001
CPR	45 (1.7)	6 (0.5)	39 (2.5)	*p* < 0.001

EMT, emergency medical technician; SBP, systolic blood pressure; DBP, diastolic blood pressure; RR, respiratory rate; HR, lower heart; LMA, laryngeal mask airway; CPR, cardiopulmonary resuscitation.

**Table 2:** In-hospital clinical parameters of younger and elderly patients.

Characteristics	Total Patient	Age ≥ 65 Years	Age < 65 Years	*p*-Value
Triage, *n* (%)				*p* < 0.001
I	192 (7.1)	55 (4.8)	137 (9.0)	
II	805 (30.0)	339 (29.5)	466 (30.3)	
III	1680 (62.5)	754 (65.6)	926 (60.2)	
IV	11 (0.4)	2 (0.1)	9 (0.5)	
Trauma team activate, *n* (%)	223 (8.3)	53 (3.4)	170 (14.8)	*p* < 0.001
In-hospital vital sign, mean ± SD				
SBP	143.4 ± 39.2	158.5 ± 33.4	132.1 ± 39.4	*p* < 0.001
DBP	82.4 ± 21.3	85.2 ± 17.1	80.3 ± 23.6	*p* < 0.001
RR	18.6 ± 4.0	19.0 ± 3.7	18.4 ± 4.1	*p* < 0.001
HR	83.2 ± 24.7	82.3 ± 25.1	84.0 ± 24.4	0.094
Shock index, mean ± SD	0.53 ± 0.18	0.54 ± 0.18	0.65 ± 0.19	*p* < 0.001
Shock index >1, *n* (%)	64 (2.4)	53 (4.6)	11 (0.7)	*p* < 0.001
Past history, *n* (%)	1001 (37.2)	449 (39)	552 (35.9)	*p* < 0.001
Cerebrovascular disease	104 (3.8)	65 (5.6)	42 (2.7)	*p* < 0.001
Coronary artery disease	613 (22.8)	362 (31.4)	251 (16.3)	*p* < 0.001
Respiratory disease	47 (1.8)	22 (1.9)	25 (1.6)	0.555
Gastrointestinal diseases	31 (1.2)	13 (1.1)	18 (1.2)	1.000
Genitourinary diseases	43 (1.6)	23 (2.0)	20 (1.3)	0.162
Malignancy	40 (1.5)	18 (1.6)	22 (1.4)	0.750
Metabolic disease	299 (11.1)	131 (11.4)	168 (10.9)	*p* < 0.001
Emergency treatment, *n* (%)				
Endotracheal tube insertion	55 (2.0)	15 (1.3)	40 (2.6)	0.026
CPR	53 (2.0)	36 (3.1)	17 (1.1)	0.124
Chest tube insertion	18 (0.7)	4 (0.3)	14 (0.9)	0.096
Cricoidectomy	1 (0.0)	0 (0)	1 (0.1)	1.000

SBP, systolic blood pressure; DBP, diastolic blood pressure; RR, respiratory rate; HR, lower heart; CPR, cardiopulmonary resuscitation.

**Table 3:** Image studies of younger and elderly patients.

Clinical Image Study	Age ≥ 65 Years	Age < 65 Years	*p*-Value
Computed tomography, *n* (%)	310 (27.0)	400 (26.0)	*p* < 0.001
Head and neck	195 (17.0)	217 (14.1)	0.031
Chest	35 (3.0)	49 (3.2)	0.911
Abdomen	12 (1.0)	24 (1.6)	0.309
Pelvis	22 (1.9)	19 (1.2)	0.154
Spine	6 (0.5)	7 (0.5)	0.786
Whole body	40 (3.5)	84 (5.5)	0.019
Angio-intervention, *n*	2 (0.2)	3 (0.2)	1.000
Magnetic resonance imaging, *n*	25 (2.2)	19 (1.2)	1.000

**Table 4:** Clinical outcome of younger and elderly patients.

Clinical Outcome	Age ≥ 65 Years	Age < 65 Years	*p*-Value
RTS, mean ± SD	7.50 ± 1.40	7.52 ± 1.29	0.678
ISS, mean ± SD	9.82 ± 11.41	9.24 ± 10.72	0.178
TRISS, mean ± SD	0.94 ± 0.18	0.94 ± 0.17	0.498
NISS, mean ± SD	10.64 ± 12.47	9.98 ± 11.80	0.163
Hospital time, mean ± SD	9.05 ± 10.86	9.04 ± 10.54	0.983
ICU admission, *n*	212	275	0.723
Re-admission ICU, *n*	6	6	0.772
ICU admission time, mean ± SD	1.45 ± 4.56	1.35 ± 4 .40	0.540
Operation, *n*	749	1000	0.967
Re-operation, *n*	44	64	0.692
Death, *n*	49	63	0.846

RTS, revised trauma score; ISS, injury severity score; TRISS, trauma injury severity score; NISS, new injury severity score; ICU, intensive care unit.

**Table 5:** Logistic regression analysis of clinical parameters between the younger and elderly patient groups.

Variable	Total Patients			Age < 65 Years			Age ≥ 65 Years		
	OR	95% CI	*p*-Value	OR	95% CI	*p*-Value	OR	95% CI	*p*-Value
Gender									
Female	-	-	-	-	-	-	-	-	-
Male	0.856	0.586–1.250	0.421	1.839	1.109–3.049	0.018	0.644	0.363–1.142	0.132
Age									
Age <65	-	-	-	-	-	-	-	-	-
Age >65	0.997	0.989–1.005	0.521	-	-	-	-	-	-
Pre-hospital									
SBP	0.994	0.985–1.004	0.236	0.994	0.980–1.009	0.452	0.992	0.978–1.007	0.308
DBP	0.981	0.996–0.995	0.010	0.979	0.960–0.997	0.026	0.984	0.961–1.007	0.172
RR	1.042	0.931–1.167	0.471	1.054	0.924–1.202	0.433	1.014	0.825–1.248	0.892
HR	0.997	0.981–1.013	0.705	0.989	0.968–1.011	0.323	1.007	0.983–1.031	0.559
In-hospital									
SBP	1.000	0.995–1.005	0.937	1.000	0.993–1.006	0.941	1.001	0.992–1.009	0.909
DBP	1.001	0.992–1.011	0.766	1.000	0.989–1.011	0.993	1.004	0.987–1.022	0.618
RR	1.007	0.963–1.053	0.759	0.995	0.938–1.056	0.871	1.016	0.968–1.066	0.520
HR	1.003	0.997–1.009	0.329	1.001	0.991–1.012	0.852	1.004	0.997–1.010	0.253
Triage, *n* (%)									
I	-	-	-	-	-	-	-	-	-
II	1.325	0.547–3.209	0.533	1.491	0.501–4.438	0.473	1.057	0.232– 4.816	0.943
III	1.408	0.604–3.282	0.428	1.462	0.514–4.157	0.476	1.251	0.293–5.351	0.762
GCS score	1.040	0.948–1.141	0.404	0.996	0.907–1.093	0.926	1.340	0.886–2.029	0.166
CT scan	0.885	0.601–1.303	0.536	0.925	0.553–1.549	0.767	0.835	0.464–1.501	0.546
Shock index	1.383	0.573–3.338	0.470	1.147	0.319–4.125	0.834	1.818	0.565–5.848	0.316
Shock index >1	1.681	0.718–3.940	0.232	1.759	0.683–4.534	0.242	1.499	0.194–11.581	0.698
Score systems									
RTS	0.469	0.428–0.513	<0.001	0.442	0.386–0.506	<0.001	0.494	0.438–0.558	<0.001
ISS	1.179	1.146–1.214	<0.001	1.210	1.161–1.261	<0.001	1.150	1.108–1.193	<0.001
TRISS	1.114	1.099–1.130	<0.001	1.111	1.091–1.132	<0.001	1.119	1.095–1.143	<0.001
NISS	0.002	0.001–0.004	<0.001	0.001	0.001–0.004	<0.001	0.003	0.001–0.008	<0.001

OR, odds ratio; CI, confidence interval; SBP, systolic blood pressure; DBP, diastolic blood pressure; RR, respiratory rate; HR, lower heart; GCS, Glasgow coma scale; CT, computed tomography; RTS, revised trauma score; ISS, injury severity score; TRISS, trauma injury severity score; NISS, new injury severity score.
